# Influence of Layer Thickness and Shade on the Transmission of Light through Contemporary Resin Composites

**DOI:** 10.3390/ma17071554

**Published:** 2024-03-28

**Authors:** Markus Heyder, Stefan Kranz, Julius Beck, Marlene Wettemann, Christoph-Ludwig Hennig, Ulrike Schulze-Späte, Bernd W. Sigusch, Markus Reise

**Affiliations:** 1 Department of Conservative Dentistry and Periodontology, Center of Dental Medicine, Jena University Hospitals, 07743 Jena, Germany; markus.heyder@med.uni-jena.de (M.H.); julius.beck@med.uni-jena.de (J.B.); markus.reise@med.uni-jena.de (M.R.); 2 Department of Orthodontics, Center of Dental Medicine, Jena University Hospital, 07743 Jena, Germany; christoph-ludwig.hennig@med.uni-jena.de; 3 Section of Geriodontics, Department of Conservative Dentistry and Periodontology, Center of Dental Medicine, Jena University Hospitals, 07743 Jena, Germany; ulrike.schulze-spaete@med.uni-jena.de

**Keywords:** nanocomposite, photopolymerization, shade, layer thickness, MARC PS, light transmission

## Abstract

Background: Material-dependent parameters have an important impact on the efficiency of light polymerization. The present in vitro study aimed to investigate the influence of the increment thickness and shade of nano- and nanohybrid resin composites on the transmission of curing light. Methods: Three contemporary resin composites were evaluated: Tetric EvoCeram^®^ (TEC); Venus Diamond^®^ (VD); and Filtek Supreme XTE^®^ (FS XTE). Light transmission (LT) was recorded in accordance with the sample thickness (0.5 to 2.7 mm) and the shade. Polymerized samples were irradiated for 10 s each using the high-power LED curing light Celalux 2 (1900 mW/cm^2^). LT was simultaneously recorded using the MARC Patient Simulator (MARC-PS). Results: LT was strongly influenced by the composite layer thickness. For 0.5 mm-thick samples, a mean power density of 735 mW/cm^2^ was recorded at the bottom side. For the 2.7 mm samples, a mean power density of 107 mW/cm^2^ was measured. Only LT was markedly reduced in the case of darker shades. From A1 to A4, LT decreased by 39.3% for FS XTE and 50.8% for TEC. Dentin shades of FS XTE and TEC (A2, A4) showed the lowest LT. Conclusions: The thickness and shade of resin composite increments strongly influences the transmission of curing light. More precise information about these parameters should be included in the manufacture manual.

## 1. Introduction

Because of their improved mechanical and aesthetic properties, resin composites are the most frequently used materials in restorative dentistry [1,2].

Nevertheless, clinical application can be error-prone to some extent. In particular, insufficient light polymerization has a strong impact on material properties and clinical survival rates [3,4,5,6]. Today, photopolymerization of resin composites is usually carried out using LED-based light curing units (LCU), which also enable high degrees of conversation [7,8].

If photopolymerization is insufficient, high amounts of short-chained residual monomers can remain within the resin matrix [9,10,11]. This might cause irritation of the dental pulp [12,13], especially in deep cavities where only a thin layer of protective dentin is left [12,14,15].

Inadequately cured resin composite restorations often exhibit reduced mechanical properties, indicated by low fracture strength and low stress resistance [16,17]. Furthermore, adhesive failure [18], increased erosion, and impaired color stability might be further signs of an insufficiently carried out photopolymerization [19,20]. Frequently, the LCU-based curing process is prone to subjective handling errors too [4,21,22]. In this regard, our group recently published data showing that increased distances of the light conductor to the composite surface, as well as sub-optimal irradiation angles, have a strong influence on the amount of polymerization light delivered [23,24,25]. 

In addition to subjective errors, several material- and processing-dependent factors also play an important role in the polymerization result. These include the incremental strength and shade of the resin composite that professionals are often unaware of in daily practice [26,27,28]. 

In this context, the respective manufacturer information on light curing parameters regarding the increment thickness and composite shade is still missing. To the best of our knowledge, there are limited data available which focus on these issues.

Therefore, the present in vitro study was aimed to investigate the transmission of light through nano- and nanohybrid composites obtained from different manufacturers, using the MARC Patient Simulator (MARC PS). Alongside the influence of the resin composite layer thickness, the effects of different shades were examined. The subject of the present in vitro study was to quantify the material-dependent properties to further improve the photopolymerization of resin composites in daily clinical practice. 

## 2. Materials and Methods

### 2.1. Test Samples

In the present in vitro study, the influence of the layer thickness (increment strength) and the shade of different resin composites on the light transmission was investigated. The following materials were analyzed: TetricEvoCeram—TEC (Ivoclar Vivadent, Schaan, Liechtenstein); Venus Diamond—VD (Heraeus Kulzer, Hanau, Germany); and Filtek Supreme XTE—FS XTE (3M ESPE, St. Paul, MN, USA). From each manufacturer, packable resin composites in the universal shades A1, A2, A3, A4, B2, B3, C2, and C3 were used. In addition, opaque dentin (A2, A4, OD, and OL) and flowables (A2 and A4) have also been investigated (Table 1 and Table 2).

At first, cylindrical test specimens of 7.8 mm in diameter and different heights (0.5, 0.7, 0.9, 1.1, 1.3, 1.5, 1.9, 2.3, and 2.7 mm) were fabricated for all packable resin composites in the universal shades A1, A2, A3, and A4 using silicone molds. In the case of the universal shades B2, B3, C2, and C3, cylindrical test samples of 1.1 mm in height and 7.8 mm in diameter were produced. Identical dimensions (7.8 × 1.1 mm) were also applied to all opaque dentin samples and flowable applications. A summary of all fabricated specimens is shown in Table 2. 

After application of the resin composites to the silicone molds, all samples were pre-cured for 40 s each using the LCU Celalux 2^®^ (VOCO GmbH, Cuxhaven, Germany) at maximum power. Subsequently, final polymerization was carried out for 180 s using the Dentacolor XS light curing device (Heraeus Kulzer, Wahrheim, Germany). The function of both curing devices was checked and calibrated in advance. For each composite height and shade, two test specimens were fabricated.

### 2.2. Light Transmission Measurement

For recording the light transmission as a function of the composite shade and increment thickness, all polymerized test specimens were centered above the anterior sensor of the MARC PS (MARC Patient Simulator, Blue Light Analytics, Halifax, NS, Canada) using a customized plastic bar. This application ensured secure positioning and fixation of the Celalux 2 LCU at a right angle in close contact with the fabricated composite specimens. 

The MARC PS is a dummy head equipped with a light sensor, 3.9 mm in diameter, located in the second quadrant between the upper two incisors at the facial side. The sensor is connected to a spectrometer (USB4000, Ocean Optics, Dunedin, FL, USA) via a fiber-optic cord. The MARC PS enables the recording of the average and maximum power (mW/cm^2^), the light fluence (J/cm^2^), and wavelength delivered by an LCU, and was originally developed for teaching, training, and calibrating purposes. 

Measurements were performed for 10 s each at maximum power of the LCU (1000 mW/cm^2^). Prior to any tests, the performance of the LCU and light sensor of the MARC PS were checked and calibrated. After every 10 runs, the light constancy and sensor function were evaluated. Here, an average power density of 1900 ± 66 mW/cm^2^ was recorded. All measurements were repeated five times.

### 2.3. Statistical Analysis

The statistical analysis was performed using SPSS version 22.0 (IBM, Armonk, NY, USA). For significance testing, variance analysis was carried out based on a linearly mixed model corrected by Bonferroni correction. The significance level was *p* < 0.05.

## 3. Results

The present in vitro study investigated the transmission of light through three contemporary resin composites of different sample thicknesses (0.5 to 2.7 mm) and shades using the MARC PS. 

**Figure 1:**
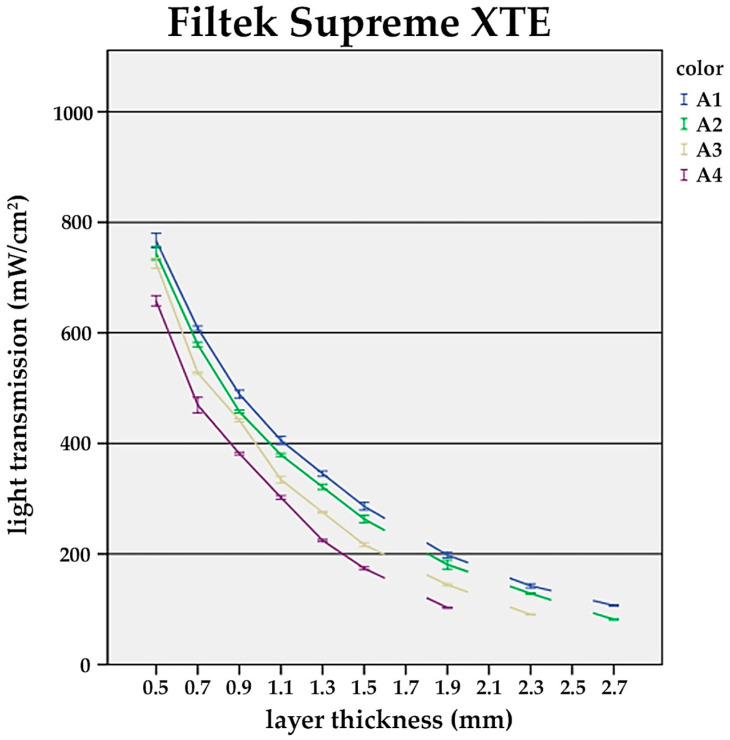
Influence of the layer thickness on the light transmission of Filtek Supreme XTE. Light transmission of the shades A1, A2, A3, and A4 at layer thicknesses of 0.5, 0.7, 0.9, 1.1, 1.3, 1.5, 1.9, 2.3, and 2.7 mm. At layers >1.5 mm, there is significant reduction in light transmission compared to 0.5 mm (*p* < 0.05).

**Figure 2:**
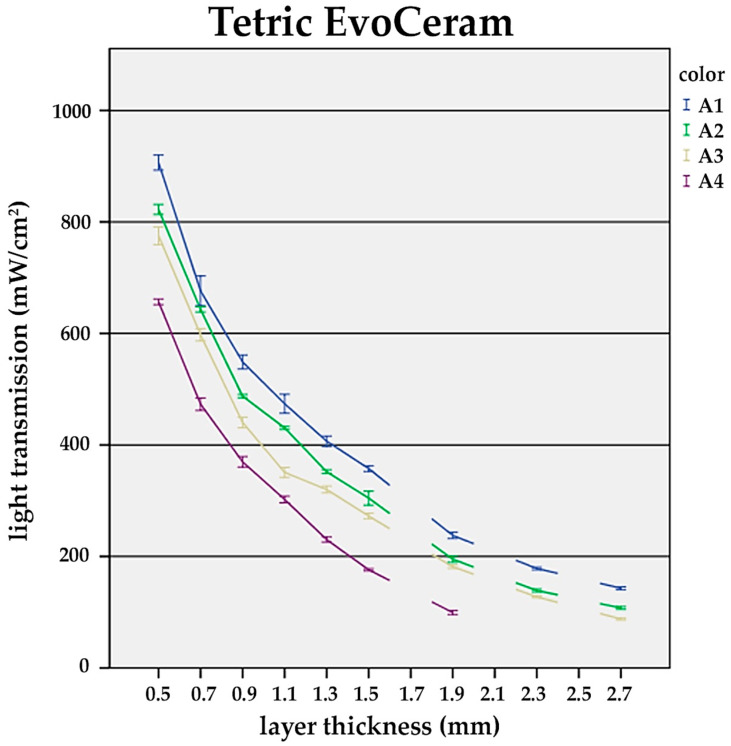
Influence of the layer thickness on the light transmission of Tetric EvoCeram. Light transmission for the shades A1–A4 and sample thicknesses from 0.5 to 2.7 mm. At layers >1.5 mm, there is significant reduction in light transmission compared to 0.5 mm (*p* < 0.05).

**Figure 3:**
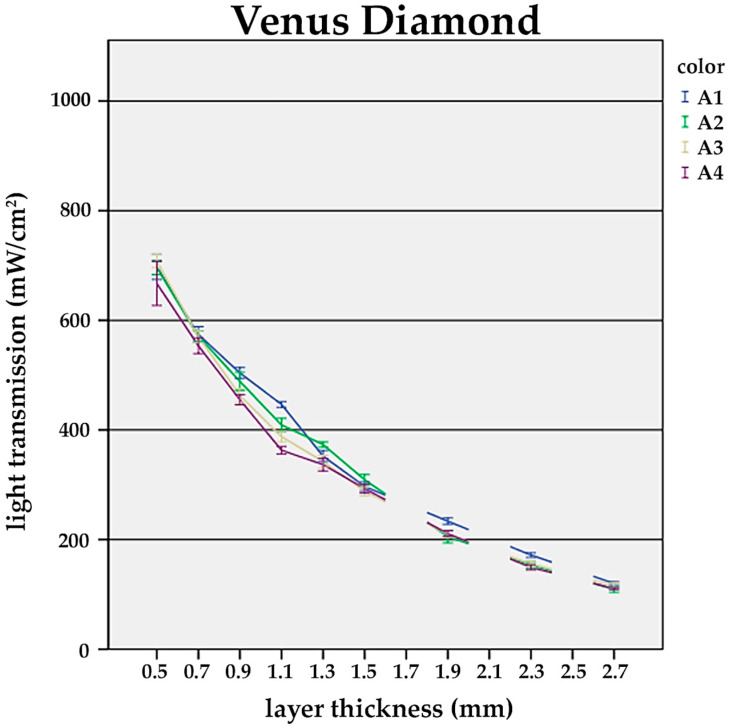
Influence of the layer thickness on the light transmission of Venus Diamond. Light transmission of the shades A1, A2, A3, and A4 at layer thicknesses of 0.5, 0.7, 0.9, 1.1, 1.3, 1.5, 1.9, 2.3, and 2.7 mm. At layer thicknesses of 1.5 mm or greater, there is a significant reduction in light transmission compared to 0.5 mm (*p* < 0.05). There was no significant difference between the shades A1 and A4.

It was found that with increasing layer thickness, the recorded power densities at the bottom side of the composite specimens decreased distinctively among the tested universal shades (A1–A4). The difference between the shades became significant at layer thicknesses ≥1.5 mm (Figure 1, Figure 2 and Figure 3). 

At a layer thickness of 0.5 mm, a mean transmission of 735 mW/cm^2^ was analyzed at the bottom side of the sample. In detail, for FS XTE, TEC, and VD, values of 735, 790, and 692 mW/cm^2^ were recorded. Transmission of light through the 0.5 mm-thick samples already caused a decrease in the original LCU power by a mean of 61.3%. 

At a layer thickness of 1.5 mm, the mean transmission value decreased to 466 mW/cm^2^ (FS XTE: 489 mW/cm^2^; TEC: 512 mW/cm^2^; VD: 396 mW/cm^2^). 

For the 2.7 mm-thick samples, the power density at the bottom side did not exceed an average of 81 mW/cm^2^ (FS XTE: 47 mW/cm^2^; TEC: 84 mW/cm^2^; VD: 113 mW/cm^2^). This correlates with only 4.3% of the light energy originally emitted by the LCU (1900 mW/mm^2^).

It was also found that for FS XTE in shade A4, the manufacturer-recommended light fluence (8 J/cm^2^) was obtained for layers less than 0.9 mm in thickness only. For TEC in shade A4, the recommended fluence (10 J/cm^2^) was recorded for layers up to a thickness of 0.7 mm. In the case of the brighter shades, the present investigation revealed that the suggested energy density at the cavity floor was attained only up to layer thicknesses of 0.7 mm when a curing time of 20 s was applied. For VD in shade A4, the recommended value (22 J/cm^2^) was not reached, even for the smallest layer applied (0.5 mm).

Alongside the layer thickness, the influence of the composite shade on the light transmission was evaluated. In general, darker shades showed distinctly lower transmission values (A1—bright; A4—dark) (Figure 1, Figure 2 and Figure 3).

For TEC and FS XTE, at a constant layer thickness of 1.5 mm, a significant drop in the power density from the brightest (A1) to the darkest shade (A4) was observed. In detail, TEC transmission decreased by 50.8%, while for FS XTE, 39.3% was recorded (Figure 1 and Figure 2). On the contrary, VD showed similar light transmission values for all A-shades (Figure 3).

**Figure 4:**
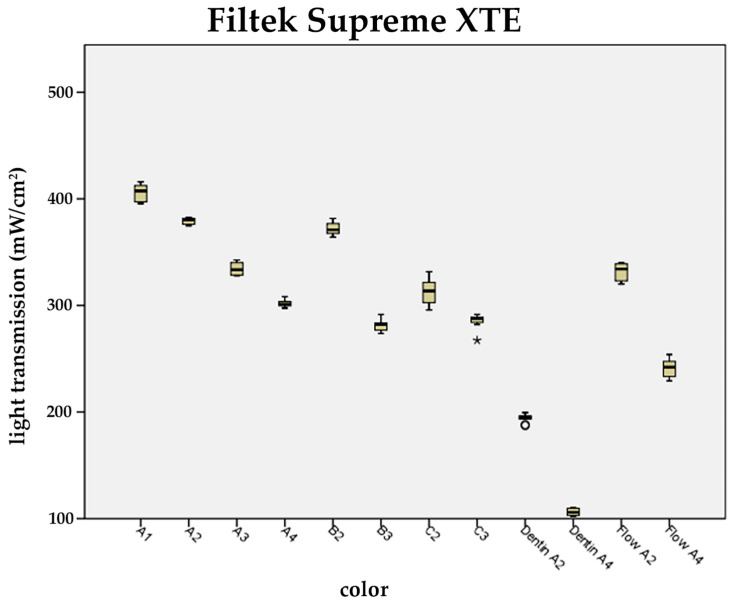
Influence of the composite shade on the light transmission of Filtek Supreme XTE. Light transmission of the universal, dentin, and flowable shades at the layer thickness 1.1 mm (universal shades: A1, A2, A3, A4, B2, B3, C2, and C3; dentin shades: A2 and A4; flowables: A2 and A4). There is a significant reduction in light transmission for the dentin colors A2 and A4. Outliers are shown as circles.

**Figure 5:**
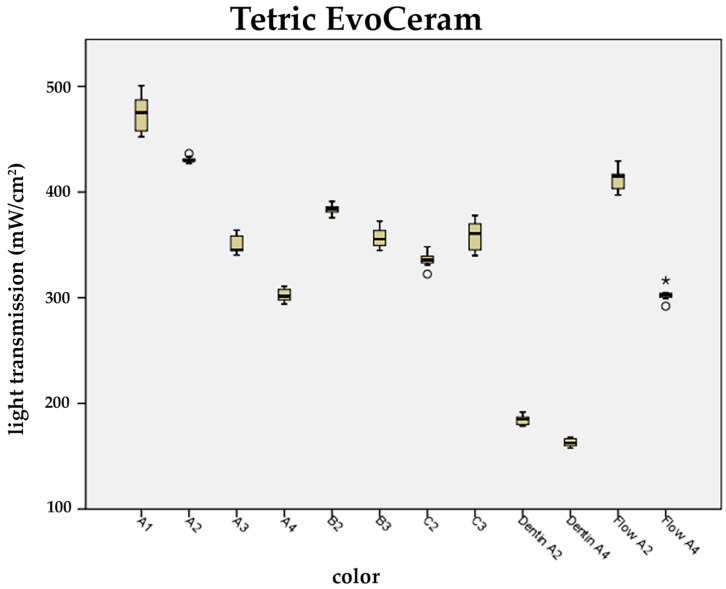
Influence of the composite shade on the light transmission of Tetric EvoCeram. Light transmission of the universal, dentin, and flowable shades at a layer thickness of 1.1 mm (universal colours: A1, A2, A3, A4, B2, B3, C2, and C3; dentin colours: A2 and A4; flowables: A2 and A4). There is a significant reduction of light transmission for the dentin colors A2 and A4. Outliers are shown as circles.

**Figure 6:**
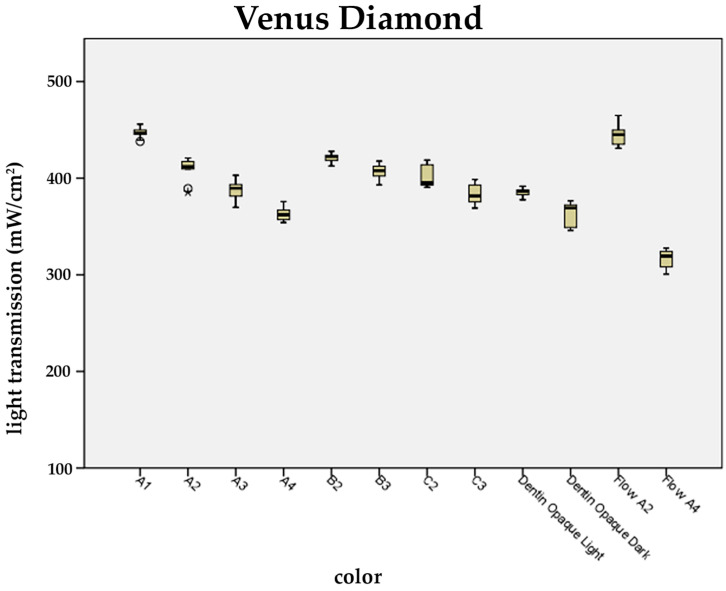
Influence of the composite shade on the light transmission of Venus Diamond. Light transmission of the universal, dentin, and flowable shades at a layer thickness of 1.1 mm (universal shades: A1, A2, A3, A4, B2, B3, C2, and C3; dentin shades OL and OD; flowables: A2 and A4; OL: opaque Light; OD: opaque dark). Outliers are shown as circles.

It was ascertained that among the universal shades B and C, and in the case of the chosen packable dentin and flowable applications, the brightest materials also showed the highest light transmission values (Figure 4, Figure 5 and Figure 6).

In the case of A2, B2, and C2, a significant decrease in light transmission from A2 to C2 was observed for packable resin composites of TEC and FS XTE only.

For FS XTE and TEC, light transmission through opaque dentin (A2, A4) at a constant layer thickness of 1.1 mm was found to be significantly decreased compared to the universal shades (A1–A4) available from the same manufacturer (Figure 4, Figure 5 and Figure 6).

Among the flowable resin composites, the brighter shades (A2) showed significantly higher light transmission compared to shade A4. An average difference in light transmission between A2 and A4 of 110 mW/cm^2^ was recorded (Figure 4, Figure 5 and Figure 6).

## 4. Discussion

The present in vitro study investigated the transmission of light at wavelengths between 430 and 490 nm through contemporary resin composites. The transmitted amount of light was recorded as a function of the increment thickness and composite shade. For measurements, the MARC Patient Simulator (MARC PS) was used. 

As shown by the results, an increase in sample thickness from 0.5 to 2.7 mm resulted in a significant drop in transmitted light by an average of 89%. It was found that the reduction was greatest for FS XTE (93.5%) and lowest for VD (83.6%). 

In this regard, it is generally suggested that under clinical conditions, composite increments should not exceed a thickness of 2 to 3 mm in order to ensure sufficient curing results [28,29]. The impact of the increment thickness on the degree of conversation is still a matter of debate [30,31,32,33].

As shown by the results of the present investigation, only 4.3% of the originally applied light fluence was recorded on the bottom side of the 2.7 mm-thick samples. Furthermore, it was found that the manufacturer’s recommended power densities were obtained for layers that did not exceed 0.9 mm in thickness only. In this context, it is generally ascertained that an increase in material volume is always accompanied by significant absorption and scattering of light, which results in reduced transmission values and therefore diminished conversation rates [34,35].

The present study further investigated the influence of the composite shade on the transmitted amount of light. Overall, eight different universal shades (packable resin composite) and two different shades of packable dentin and flowable applications were evaluated (Table 2). 

As shown by the results, light transmittance was lower for darker shades. In detail, between the shades A1 and A4, a drop of 39.3% for FS XTE and 50.8% for TEC was recorded. A reduction in transmission was also obtained for the B- and C-type shades. For FS XTE and also TEC, differences between B2 and B3, as well as C2 and C3, were found to be significant. In the case of VD, there was no significant difference in light transmission between the shades A1 and A4. This might be due to the use of larger filler particles that, in general, cause less scattering of light. In this regard, the applied color pigments might only have a minor impact on the transmission of light. 

But in general, the composite shade has a strong impact on the transmission of light [36]. Further, it was proven that under identical curing conditions, composites of darker shades are afflicted by lower conversation rates compared to brighter materials [25,37,38]. 

In the present study, it was also proven that for identical shades and sample thicknesses, light transmission differed among the single manufacturers. In detail, VD, almost throughout, showed the highest transmission values. Despite of identical product names, resin composites from different sources may nevertheless vary in transmission too. In order to ensure sufficient curing results, an adoption of the recommended curing times is therefore required [39]. The transmission of light through resin composite is influenced by several factors, such as the filler type, filler-to-resin ratio, and the organic matrix. Each component, further, has its own specific refractive index [40,41,42]. This might be some of the reasons that explain the diversity of transmission values observed among the tested materials in the present investigation.

As already known, small filler particles, which are usually incorporated in micro- and hybrid-type resin composites, cause extensive scattering of light and thus diminished transmission values [26,43]. In the present study, the most favorable transmission of light was observed for samples manufactured from VD. This is probably due to the content of bigger filler particles that can reach values of up to 20 µm in size. 

The examined nano- and nanohybrid-type resin composites also significantly differed among their organic matrices. While FS XTE and TEC contain conventional monomers such as BisGMA, BisEMA, UDMA, and TEGDMA, VD is composed of a tricyclodecan–urethan matrix instead (Table 1). Differences in the refractive indices between inorganic filler particles and the organic matrix might also be a reason for the lower light transmission of FS XTE and TEC [44,45,46].

The results of the study also demonstrate that packable opaque dentin applications are afflicted by lower light transmittance too. In particular, FS XTE and TEC showed significantly reduced values by 150 mW/cm^2^ and 174 mW/cm^2^ when compared to the brighter universal shades of identical thickness. Other authors, too, noted that photopolymerization is more efficient among universal shades as compared to opaque materials [47,48]. It can be assumed that the general recommended maximum of 2 mm in layer thickness needs to be adjusted in accordance with the applied shade and type of resin composite. In particular, it was realized that the opacity has a strong impact on the curing depth [48,49].

In the case of the flowable resin composites that were examined in the present study, transmissions values similar to, or somewhat lower than, those of the packable applications were obtained. Flowable resin composites differ from packable types mainly in terms of their filler particles content [50]. Since there are only slightly differences, packable and flowable resin composites show similar physical properties, which presumably also applies to their close light transmission values [51]. 

The required light energy for complete polymerization depends on the type of material applied. Therefore, curing parameters and power settings need to be adopted [22]. The results of the present study suggest that data sheets should be individualized in accordance with the respective shade and increment thickness. 

So far, a fluence of 8 J/cm^2^ is recommended by the manufacturer for FS XTE and 10 J/cm^2^ for TEC, independently of the shade employed. In the case of VD, the manufacturer recommends 11 J/cm^2^ for the brighter shades (A1–A3) and 22 J/cm^2^ for A4.

However, the LCU that was used in the present investigation delivered a maximum power density of 1900 mW/cm^2^ and was thus well above the manufacturer’s specifications (1000 mW/cm^2^). Operation for 20 s caused a light fluence of 38 J/cm^2^ directly at the sensor surface. However, the results of the present study have shown that light curing for 20 s was efficient in delivering the required amount of energy to the cavity floor, but only for thin layers and brighter shades. In detail, it was found that for FS XTE in shade A4, the required light fluence (8 J/cm^2^) was achieved only for layers less than 0.9 mm in thickness. For TEC in shade A4, the recommended fluence (10 J/cm^2^) was obtained for layers up to 0.7 mm only. As already mentioned above, for VD, the suggested light fluence is 11 J/cm^2^ for brighter shades (A1–A3) and 22 J/cm^2^ for the shade A4. 

However, in the case of the brighter shades, the present investigation revealed that the suggested energy density at the cavity floor was attained only up to layer thicknesses of 0.7 mm when a curing time of 20 s was applied. For VD in shade A4, the recommended value was not reached, even for the smallest layer applied (0.5 mm).

Curing time has a significant impact on the material properties, such as degree of conversation, knoop hardness, and elastic modulus. As recently observed, exposure in the range between 24 and 48 J/cm^2^ is more efficient compared to the delivery of 12 or 16 J/cm^2^ [52]. In this regard, it was suggested that the survival of dental restorations within the oral cavity can be extended due to the use of longer photopolymerization durations [22]. But photocuring also has a significant thermal aspect. It was shown that alongside higher degrees of conversation, longer exposure times were also afflicted by increased temperature values. The highest temperature ranges (up to 50 °C) were observed when high-intensity laser devices were used for curing [53].

It is also well known that irradiation time needs to be adapted in accordance with the increment thickness. To prevent the cytotoxic effects that can arise from unpolymerized monomers, irradiation time needs to be expanded in the case of thicker increments. In this regard, it was shown that from photopolymerized 2 mm-thick resin composite increments, fewer monomers, such as TEGDMA, Bis-GMA, and UDMA, were released compared to thicker layers that were identically cured [54].

By considering the results of the present investigation, it is suggested to expend the curing time (>20 s) in accordance with the composite shade and layer thickness. The problem of inadequate manufacturer recommendations is also discussed by other authors [55,56]. Altogether, the results of the present in vitro study indicate that it is important to analyze curing parameters in detail in order to ensure more sufficient photopolymerization. 

## 5. Conclusions

Light transmission through nano- and nanohybrid composites is significantly influenced by increment thickness and material shade. 

In particular, the thickness of the resin composite increments has a strong impact on the curing results since light transmission distinctly decreases with increasing layer thickness.

In order to ensure sufficient light polymerization, especially for bigger increments and/or darker shades, expanded curing times should be recommended by the manufacturer. In adjusting the curing time, it is suggested to take the layer thickness, composite shade, and manufacturer into account.

## Figures and Tables

**Table 1:** Material composition.

Manufacturer	Composite	Composition
3M ESPE, St. Paul, MN, USA	Filtek Supreme XTE	Filler: 72.5 wt.% (55.6 vol.%); zirconium oxide/silicon dioxide cluster (0.6–10 µm) consisting of 20 nm silicon and 4–11 nm zirconium particles, non- agglomerized/non-aggregated silicon nanofillers (20 nm), and zirconium nanofillers (4–11 nm) Matrix: Bis-GMA, BisEMA, UDMA, TEGDMA, PEGDMA
	Filtek Supreme XTE Flowable	Filler: 65 wt.% (46 vol.%); zirconium oxide/silicon dioxide cluster (0.6–10 µm) consisting of 20 nm silicon, 4–11 nm zirconium particles, ytterbium trifluoride (0.1–0.5 µm), non-agglomerized/non-aggregated surface-modified 20 nm silica filler, and 75 nm silica filler Matrix: Bis-GMA, Bis-EMA, TEGDMA
Ivoclar Vivadent, Schaan, Liechtenstein	Tetric EvoCeram	Filler: 75–76 wt.% (53–55 vol.%) inorganic fillers; barium glass, ytterbium trifluoride, mixed oxide (particle size of the inorganic fillers 40 nm–3000 nm, mean size 550 nm) and prepolymer (34 wt.%) Matrix: BisGMA, UDMA, ethoxylated Bis-EMA
	Tetric EvoFlow	Filler: 57. wt.% (30.7 vol.%) inorganic fillers; barium glass, ytterbium trifluoride, high-dispersion silicon dioxide, mixed oxide (particle size of the inorganic fillers 40 nm–3000 nm, mean size 550 nm), and prepolymer (20.4 wt.-%) Matrix: Bis-GMA, UDMA, DDDMA
Heraeus Kulzer, Hanau, Germany	Venus Diamond	Filler: 80–82 wt.% (64 vol.%); barium–aluminium fluoride glass, discrete nanoparticles (particle size: 5 nm–20 µm) Matrix: TCD-DI-HEA, UDMA
	Venus Diamond Flow	Filler: 65 wt.% (41 vol.%); barium–aluminium fluoride silicate glass, ytterbium trifluoride, and silicon dioxide (particle size: 20 nm–5 µm) Matrix: UDMA, EBADMA

**Table 2:** Summary of the resin composite shades (1.1 mm-thick samples).

Composite	Universal	Dentin	Flowable
Filtek Supreme XTE	A1; A2; A3; A4 B2; B3 C2; C3	A2; A4	A2; A4
Tetric EvoCeram	A1; A2; A3; A4 B2; B3 C2; C3	A2; A4	A2; A4
Venus Diamond	A1; A2; A3; A4 B2; B3 C2; C3	OL; OD	A2; A4

## Data Availability

Data can be obtained from the corresponding author on request.
